# Dietary Iron Sources Among 9-Month-Old Infants from Low-Income Households

**DOI:** 10.3390/nu18091417

**Published:** 2026-04-30

**Authors:** Elizabeth F. Acquah, Jeffrey D. Labban, Seth M. Armah, Maureen M. Black, Marjorie Jenkins, Deborah Clarice Andoh, Jigna M. Dharod

**Affiliations:** 1 Department of Nutrition, School of Health and Human Sciences, University of North Carolina at Greensboro, Greensboro, NC 27412, USA; efacquah@uncg.edu (E.F.A.); s_armah@uncg.edu (S.M.A.); dcandoh@uncg.edu (D.C.A.); 2 College of Education, Health and Human Sciences, University of Tennessee, Knoxville, TN 21201, USA; jlabban@utk.edu; 3 Department of Pediatrics, University of Maryland School of Medicine, Baltimore, MD 27401, USA; mblack@som.umaryland.edu; 4 Nursing Research Unit, Cone Health, Greensboro, NC 27401, USA; marjorie.jenkins@conehealth.com

**Keywords:** iron, infant feeding, iron-fortified cereal, dietary iron

## Abstract

**Background**: The 2025–2030 Dietary Guidelines for Americans recommend that 6–12-month-old infants receive 11 mg iron/day. The contribution of iron-rich foods in meeting guidelines is unclear. **Objectives**: The aims were to: (1) determine the contribution of iron-fortified cereal, infant formula and heme-iron sources to infants’ total dietary iron intake; (2) examine differences in iron adequacy by milk-feeding type; and (3) identify feeding patterns associated with meeting daily iron requirements through dietary sources. **Methods**: Mothers of infants were recruited from a pediatric clinic and 24 h feeding recalls were conducted to estimate infants’ iron intake. Infants’ milk-feeding types were: breastmilk only (BF), mixed (MF), or infant formula only (FF). Main outcomes were: meeting/not meeting daily iron requirement (11 mg) overall and by milk-feeding type; contribution of iron-fortified infant cereal, formula and meat to daily iron intake. Descriptive statistics, bivariate chi-square tests, and multivariate logistic regression analyses were conducted. **Results**: Most participants identified as African American or Hispanic (76%) and were enrolled in the Special Supplemental Nutrition Program for Women, Infants, and Children (84%). Thirty-nine percent consumed < 11 mg iron/day from dietary sources. By milk-feeding type, inadequate iron intake was significantly higher among the BF (72%) and MF (74%) groups vs. the FF group (24%, *p* < 0.05). Iron-fortified cereals were consumed by 46% of infants and provided a median iron intake of 6.75 mg. Among the FF group, infant formula provided 63% of the daily iron requirement. **Conclusions**: Inadequate dietary iron intake is common. Iron-fortified cereal is an important dietary iron source. Future research is warranted to understand the relations among infants’ daily iron intake, iron sources (heme vs. non-heme), and iron status.

## 1. Introduction

Optimal nutrition during infancy is foundational for future health, as nutrient deficiencies during this critical developmental period can impact growth, cognition, and health throughout life. Iron deficiency is the most prevalent nutritional deficiency worldwide [1] and can lead to anemia and irreversible neurodevelopmental impairments [2]. Globally, approximately 42% of children are iron-deficient [3]. Adequate iron intake supports critical biological processes, including myelination and neurotransmitter synthesis, both of which are fundamental for brain development. Consequently, iron deficiency during infancy has been associated with cognitive delays, atypical socio-emotional behaviors, and other developmental challenges [4,5,6].

Prenatal iron stores are generally depleted by six months of age; therefore, feeding choices during the complementary feeding period play a critical role in infants’ iron status [1]. The 2025–2030 Dietary Guidelines for Americans recommend a daily iron intake of 11 mg for infants aged 6 to 12 months [5]. Based on this recommendation, results of national surveys indicate an estimated 20% of infants in the United States fail to meet daily iron requirements [7,8,9].

The Special Supplemental Nutrition Program for Women, Infants, and Children (WIC) is a federal food assistance program that provides economic support to families at risk of food insecurity, ensuring access to nutrient-rich foods and preventing deficiencies among pregnant and postpartum women, infants, and young children. The prevention of iron deficiency among infants is one of the key WIC goals [10]. WIC provides iron-rich or iron-fortified infant foods, including iron-fortified infant cereal, iron-fortified infant formula and infant meats. For example, one serving (1 tablespoon or 15 g) of iron-fortified infant cereal, as recommended by the DGA for infants aged 6–12 months, provides 6.75 mg of iron, about 60% of the recommended daily intake [11]. However, a national survey among children enrolled in WIC found that approximately 13% had not been exposed to iron-rich cereal by age 7 months [12].

Iron bioavailability is a key factor when considering dietary iron sources. Compared with iron-fortified infant formula (about 12 mg/L of iron), breastmilk contains relatively low concentrations of iron (approximately 0.2–0.4 mg/L) [13,14]. However, the bioavailability of iron from human milk is substantially higher (about 50%) than that from infant formula (approximately 12%) [6,15,16]. Despite the higher bioavailability of human milk, iron-fortified cereals and other iron-rich sources are recommended for breastfed infants to meet the increased requirement. A study examining iron absorption rate found that the mean iron absorption of 6- to 9- month-old breastfed infants was 0.27 mg/day, far less than the estimated physiologic requirement of 0.69 mg/day [13]. Heme iron sources, particularly red meat, are recommended during complementary feeding [14]. A study examining commercial infant meat options identified over 50 varieties of jarred baby foods, the majority of which were chicken- or turkey-based, with only about 20% consisting of red meat or beef [15].

To better understand dietary iron sources among infants, the aims of this study were to: (1) determine the contribution of iron-fortified cereal, infant formula and heme-iron sources to total dietary iron intake among infants; (2) examine differences in iron adequacy by milk-feeding type (breastmilk only, mixed, or infant formula only); and (3) identify feeding patterns associated with meeting recommended daily iron requirements through dietary sources, among infants eligible or participating in WIC at nine months of age. The significance was to generate evidence on the use and contribution of WIC-approved iron-rich infant foods—such as iron-fortified cereals, infant formula, and infant meats—to daily iron intake among infants during the complementary feeding period.

## 2. Materials and Methods

The study protocol was approved by the UNC Greensboro Institutional Review Board (19-0011).

### 2.1. Recruitment and Selection Criteria

Mother–infant dyads were recruited from a local pediatric clinic primarily serving families enrolled in Medicaid. Mothers were informed that study participation involved phone interviews of 24 h feeding recalls when the infants were 6, 9, and 12 months old. The inclusion criteria were that the mothers be at least 18 years of age, have a full-term infant with no food restrictions or allergy-related conditions and be enrolled in or eligible to participate in the WIC program. Mothers younger than 18 years of age and infants born at <37 weeks of gestation were excluded to reduce potential differences in living arrangements and feeding regimens typically associated with adolescent mothers and preterm infants. Recruitment was conducted in person in the clinic waiting room. Trained research assistants approached potential participants, described the study procedures, and obtained written informed consent from eligible mothers who expressed interest. Interviews were conducted in either English or Spanish by trained bilingual research assistants.

### 2.2. Data Collection

Mothers were contacted by phone to complete an interview that included a 24 h feeding recall and questions on sociodemographic characteristics, including household income and current WIC participation.

24 h Feeding Recall: The recall was conducted using the Nutrition Data System for Research (NDSR v 2020) software (Nutrition Coordinating Center, University of Minnesota, Minneapolis, MN, USA). Bilingual research assistants fluent in English and Spanish completed a two-day NDSR training and conducted 10 practice recalls prior to data collection. To improve portion size estimation, participants were sent images of standard sippy cups, bottles, and spoons one day prior to the scheduled recall. Recalls were conducted using the multiple-pass method. During the first pass, all feeding times were recorded. In the second pass, detailed information was obtained for each feeding, including the type of food or beverage, amount consumed, and preparation method. For infant cereal, the brand and type were collected (rice, oat, wheat), including the amount used (number of tablespoons or teaspoons), and the ingredient used to mix the cereal (water, milk, infant formula, juice, puree, etc.). Mothers were subsequently asked to report how much of the prepared cereal the infant consumed, and adjustments were made accordingly for net intake.

For breastmilk feeding, mothers were asked to specify whether feeding was direct or expressed. For expressed breastmilk, the amount consumed was recorded similarly to formula feeding. For direct breastfeeding, the volume was not recorded and was later estimated using a standard method, assuming an average total milk intake of 600 mL per day for infants at this age, alongside complementary foods. The amount of breastmilk per feeding episode was calculated by dividing 600 mL by the total number of feeding episodes. In mixed feeding, the reported formula intake was subtracted to adjust the estimated breastmilk volume per feeding. Expressed breastmilk intake was assessed in the same manner as infant formula, with mothers reporting the amount consumed [15].

The complete recall was reviewed aloud at the end of each interview to ensure accuracy and completeness. Prior to analysis, all recalls underwent quality checks to identify and correct potential reporting or data entry errors, particularly errors affecting portion sizes and total energy intake. The NDSR database includes nutrient composition data for over 1000 infant foods, including breastmilk, dairy and non-dairy formulas, infant cereals, meats, and other complementary foods. Based on the reported amounts, the NDSR uses standard nutrient composition values to estimate nutrient intake per mL or ounce of food consumed [16].

For this study, interviews and 24 h feeding recalls conducted with mothers when infants were 9 months of age were analyzed to represent the complementary feeding period. At 9 months, 227 of the total 240 mothers recruited in the main study participated in the interviews and 24 h feeding recalls; the remaining 13 participants either missed the call or were lost to follow-up.

### 2.3. Key Variables

For analysis, NDSR output files were utilized to determine total iron intake, including iron from breastmilk. To meet the study objectives, the iron amount from each source of interest, iron-fortified infant formula, iron-fortified infant cereal, infant meat, other meat and eggs, was calculated. Iron from breastmilk was not calculated separately. Based on the recalls, infants were categorized into three mutually exclusive milk-feeding types: (1) breastmilk only (received only breastmilk); (2) mixed: breastmilk and infant formula (received both breastmilk and infant formula); (3) formula only (received only infant formula).

### 2.4. Statistical Analysis

All analyses were conducted using IBM SPSS Statistics for Windows, Version 21.0 (Armonk, NY, USA: IBM Corp.) or R v 4.5.2 [17]. Statistical significance was set at *p* < 0.05. Descriptive statistics were used to examine sociodemographic characteristics, milk-feeding type, average iron intake, and the proportion of infants meeting (≥11 mg) or not meeting (<11 mg) the daily iron requirement. The percent contributions of infant formula and complementary foods to total daily iron intake were calculated, and frequency analyses were conducted to estimate the percent contribution from specific food items such as infant cereal, meats, and eggs. Iron from breastmilk was included in total iron intake; the specific contribution from breastmilk was not estimated separately.

Gamma regression with an identity link function was performed to assess differences in average iron intake from infant formula, cereal, and other complementary foods by milk-feeding type. Relative risk for not meeting the daily iron requirement was estimated using log-binomial models as implemented in the logbin package [18,19] for R [17]. Risk estimates were adjusted for the same-day provision of infant formula (dry, powdered form in oz) and cereal (g). Raw values for cereal were scaled, such that model coefficients should be interpreted as the mean expected change in risk of not meeting daily iron requirements per 10 g of cereal. Other theoretically relevant participant-level covariates—including infant sex, maternal education and employment, and household income—were also considered for inclusion. However, none of these variables was significantly associated with risk of not meeting the recommended daily iron requirement, nor did their exclusion alter model performance as assessed using likelihood ratio tests (LRT *p* > 0.80). Bootstrapping with 5000 replications was conducted to estimate standard errors and 95% bias-corrected accelerated confidence intervals. Statistical significance was determined by confidence bands for model estimates (i.e., prior to exponentiation) that did not include zero.

## 3. Results

Most mothers identified as African American or Hispanic (76%) and were enrolled in the WIC program (84%). As shown in Table 1, about one-quarter of participants reported a monthly household income of $1500 or less, and half of the mothers were employed either full- or part-time. Among infants, 46% were males and 38% were firstborn. Regarding milk-feeding status, 16% of infants were in the breastmilk-only group, while 15% and 69% were in mixed and formula-only groups, respectively.

**Figure 1:**
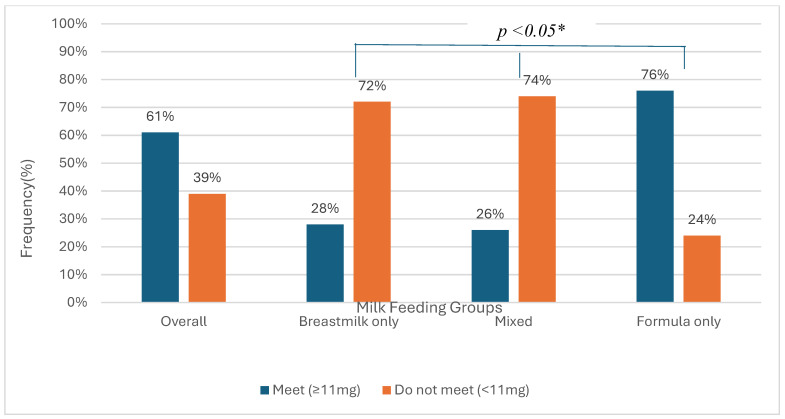
Percentage of infants that “Meet” vs. “Do not meet” the daily iron requirement through dietary sources, overall and by milk-feeding type (*n* = 224) *. * Chi-square test comparing groups by milk-feeding type.

Most infants (61%) met the recommended daily iron intake of 11 mg (Figure 1). When examined by feeding type, a significant difference was seen. As shown in Figure 1, 76% of infants in the formula-only group met the daily iron intake recommendation. In comparison, only 28% of infants in the breastmilk-only group and 26% in the mixed group met the recommendation (*p* < 0.05).

In examining the contribution of iron-fortified infant cereal and animal-iron sources, as shown in Table 2, 46% of infants consumed iron-fortified infant cereal at least once the previous day, and 41% consumed meat and/or eggs. Iron-fortified infant cereal constituted a median contribution of 6.75 mg of iron to infants’ daily intake, including rice, oat, and whole wheat varieties. Median contributions of meat and egg consumption to daily iron was 0.43 mg (Table 2). Specifically, 10% of infants consumed commercial infant meat products, primarily poultry-based options (e.g., chicken with gravy, chicken and apple combinations); 16% and 20% received eggs and non-infant poultry items, respectively.

When examined by milk-feeding type, the formula group consumed an average of 17.58 mg of daily iron from dietary sources (Table 3). The breastmilk-only and mixed milk-feeding groups consumed an average of 7.21 mg and 9.72 mg of iron per day, respectively. Neither infant cereal nor meat and egg consumption varied significantly by milk-feeding type group.

Iron-fortified formula contributed 63% of the daily iron intake in the formula-only group and 47% in the mixed milk-feeding group. For infants in the breastmilk-only group, 33% consumed iron-fortified cereal and it contributed to 76% of their daily iron requirement. Among infants in the mixed group, 47% consumed iron-fortified cereal and it contributed to about half of their daily iron intake. As shown in Table 3, meat and eggs accounted for approximately 9% and 8% of daily iron intake in the breastmilk-only and mixed feeding groups, respectively, compared to an average of about 4% in the formula group.

**Figure 2:**
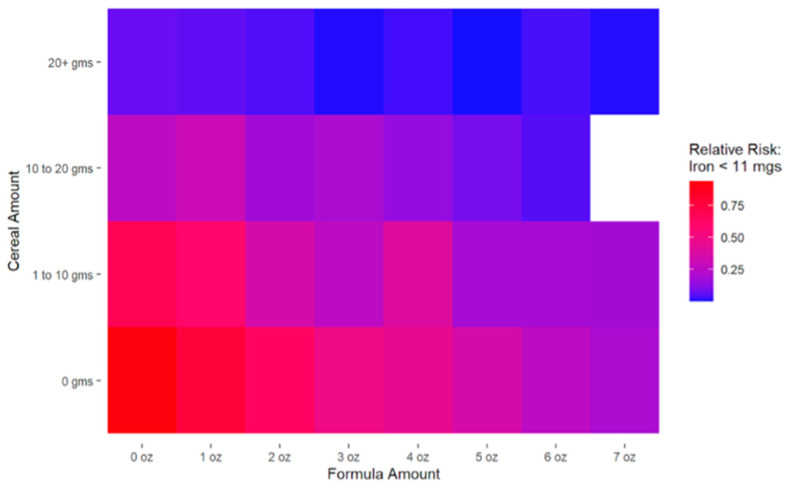
Estimated risk of inadequate iron intake by iron-fortified formula and infant cereal consumption among infants at nine months (*n* = 224). Relative Risk: Model-implied probabilities based on results from the log-binomial model. Formula Amount: in oz. in dry powdered form.

Results of the log-binomial regression model indicated that the feeding of infant formula (β = −0.209, SE = 0.030, *p* < 0.001) and infant cereal (β = −0.844, SE = 0.177, *p* < 0.001) were significantly associated with decreased risk of inadequate iron intake. The heat map (Figure 2) illustrates the joint association of infant formula and infant cereal intake with the risk of failing to meet the recommended daily iron intake (≥11 mg/day). Specifically, model-implied probabilities for missing recommended daily iron intake were calculated at different combinations of formula and cereal intake amounts. A clear gradient was seen, with the highest probability (brighter red) of inadequate iron intake occurring when infants did not consume infant cereal or infant formula. Among infants who did not receive infant formula partially or exclusively, intake of 20 g or more of iron-fortified infant cereal (more than one serving/~2 tablespoons) minimized the risk of inadequate iron intake. Conversely, among infants who consumed no infant cereal, fully formula feeding mitigated (but did not minimize) risk, with notable improvement around 5 oz of formula powder (~33 oz reconstituted).

## 4. Discussion

In this study conducted during the complementary feeding period, we found that iron-fortified infant formula and iron-fortified infant cereal were the primary contributors to infants’ total dietary iron intake. In contrast, consumption of heme-iron sources—such as commercially prepared infant meats—was relatively uncommon. Infants who were given breastmilk with or without infant formula were at higher risk of inadequate iron intake, especially when iron-fortified cereal was not given once or twice daily. A single serving of iron-fortified infant cereal (15 g or 1 tablespoon) provides approximately 60% of the recommended 11 mg of daily iron intake [20]. Similar to our study results, Abrams et al. (2021) also found infant cereal to be the major source of iron for breastfed infants [13]. Iron-fortified infant cereals are processed grain-based foods sold in a dry, powdered form and commonly available in varieties such as rice, oats, and whole wheat. Within the WIC program, formula-fed infants receive an 8 oz container of cereal, whereas infants who receive breastmilk without formula receive 16 oz to help compensate for the lower iron content of breastmilk. Iron-fortified infant cereal is frequently recommended as a first complementary food because of its nutrient density, mild flavor, and easily adjustable texture [21]. However, infant feeding studies suggest that only about half of U.S. infants consume infant cereal daily [22]. For example, analyses of NHANES data from 2001 to 2014 indicate that 47% of the infants aged 7 to 11 months were not fed infant cereal each day [23]. Similarly, in our study, about half of the infants had not consumed infant cereal at least once on the previous day. By milk type, the 2016 Feeding Infants and Toddlers Study (FITS) survey reported that 60% of exclusively breastfed infants and 41% of mixed-fed infants were not given infant cereal, which significantly increased their risk of not meeting the recommended daily iron intake [24]. Despite the high bioavailability of iron in breastmilk, its iron content is low. Infants who are partially or exclusively breastfed are recommended to receive 1 mg/kg per day of oral iron starting at four months of age, followed by the introduction of iron-rich complementary foods, including iron-fortified cereals, during the complementary feeding phase [25,26]. The reasons for low infant cereal intake are not well understood, but the growing emphasis on promoting fruits and vegetables, coupled with less focus on the importance of infant cereal, may contribute to the declining trend. While it is important to advise caregivers not to add cereal to bottles—a common practice associated with early introduction to solids and higher energy intake [26]—it is equally critical to promote infant iron-fortified cereal consumption (~2 tbsp/day) as a solid food, alongside continued support for breastfeeding.

In our study, iron-fortified infant formula was a significant source of iron among the formula-only group. A study examining serum ferritin and hemoglobin levels by milk-feeding type among 9-month-old infants found that infants who received breastmilk with or without infant formula were at a significantly higher risk of iron deficiency, concluding that iron-fortified infant formula contributed to the iron adequacy for the children examined [27]. Similar to our findings, a review of complementary feeding data in the U.S. showed that formula-fed infants consumed an average of 17 mg of iron per day, with formula being a significant source of iron in this population [28]. Most of our participants were enrolled in the WIC program. Although the requirements were temporarily adjusted during the recent formula shortage, the WIC-contracted infant formulas must contain at least 10 mg/L of iron—substantially higher than European commission and CODEX Alimentarius standards [29]. In a recent perspective, Abrams and Bergner emphasized the need to revise the Infant Formula Act of 1980, which defines the nutrient regulations for infant formulas sold in the U.S. Updating standards on iron concentrations in infant formula was highlighted, citing emerging evidence of negative associations between excessive iron intake and infants’ growth [30].

In our study, intake of infant meat was uncommon, aligning with national data. According to the 2016 FITS Survey, only 4% of infants consumed infant meat daily. Although heme iron is more bioavailable than non-heme iron, this low level of infant meat intake likely limits the overall contribution of heme iron to meeting iron requirements. Further, an analysis of iron content in infant foods found unexpectedly low levels of iron in commercial infant meats [20]. Among 74 varieties of infant meat products (including jars and mixed meals), only 3% met the criteria for a “good” source of iron (10–19% DV), and none met the threshold for an “excellent” source (≥20% DV). These low iron levels were attributed to the limited availability of single-ingredient pureed meats—particularly red meats—and the predominance of mixed dinners that combine a meat source with vegetables or starches. Similarly, in our study, infants primarily consumed combination products (e.g., apple with turkey), with very limited intake of red meat.

In contrast, egg consumption was relatively more common. In an analysis of 2001–2012 NHANES data comparing nutrient profiles and growth outcomes between infant egg consumers and non-egg consumers aged 6 to 9 months, daily egg consumption was found to be more common among infants enrolled in WIC than among non-WIC participants. Although no differences were observed in iron consumption between the two groups, infants who consumed eggs had significantly greater recumbent length with no differences in body weight [31]. In a clinical trial, infants received eggs daily for six months. At the end of the study, no significant differences in iron status or anemia prevalence were observed between the intervention and control groups, highlighting that although eggs are an animal-source food, their iron is largely bound to phosvitin—an enzyme that limits absorption—making them a limited contributor to infants’ iron status [32].

## 5. Strengths, Limitations and Future Studies

This study contributes to the growing body of evidence on dietary iron sources during the complementary feeding period among infants eligible for or participating in WIC. Our study provides information on the extent to which iron-fortified cereal can help reduce the risk of inadequate iron intake among infants, highlighting the importance of this key complementary food provided by WIC. Several limitations should also be noted. First, participants were recruited from a single pediatric clinic, which may limit the generalizability of the findings. Nonetheless, the sociodemographic characteristics of our participants closely reflect those of families participating in WIC. Second, dietary intake was based on a single 24 h recall rather than the ideal multiple recalls for a precise estimate of nutrient intake. However, the previous day’s intake reflected the infant’s usual consumption (vs. more or less than typical). It is also important to note that our study did not measure iron biomarkers such as ferritin, transferrin or hemoglobin concentration; hence, our findings are limited to iron intake adequacy and cannot be used for physiological iron sufficiency. This distinction is especially important considering that the bioavailability of iron differs significantly by source. Heme iron is more efficiently absorbed, whereas the absorption of non-heme iron is more variable and influenced by other dietary components. Iron-fortified infant cereals and infant formulas contain non-heme iron. In contrast, commercially available infant meats such as turkey and chicken baby foods provide heme iron, which, although lower in amount per serving, may serve as an important complementary source due to its higher bioavailability. Lastly, our analysis focused on dietary sources of iron and did not capture supplement use. National data indicate that fewer than 10% of WIC infants during the complementary feeding period receive any dietary supplements, most commonly multivitamins (A, C, D, E) or vitamin D drops; use of iron drops was even lower than average supplement use [32]. Hence, future studies examining sources of heme versus non-heme iron and their association with infant iron status are warranted.

## 6. Conclusions

To ensure optimal growth and development, promoting complementary feeding practices that help infants meet their daily iron requirements is critical. Hence, in addition to supporting continued breastfeeding at the program and policy levels, efforts to improve iron intake through multiple dietary sources—such as iron-fortified infant cereals and heme-iron sources including meats and poultry—are warranted. Our study showed that consuming at least 20 g (~2 tbsp) of infant cereal increased an infant’s chances of meeting the daily iron recommendation. However, further research is also needed to understand the limited use of infant meats and the potential contribution of iron from breastmilk intake. In sum, the findings from this study underscore the need for targeted nutrition education and interventions that prioritize iron-rich infant cereals, along with culturally appropriate use of both commercial and home-prepared iron-rich complementary foods, to reduce the risk of inadequate iron intake during infancy.

## Figures and Tables

**Table 1:** Characteristics of mother–infant dyads (*n* = 224).

Characteristics	*n* (%) ^1^
**Infant Sex**	
Male	104 (46)
Female	121 (54)
**Parity**	
Primiparous	86 (38)
Multiparous	139 (62)
**Race/Ethnicity ^2^**	
African American	85 (38)
Latino	86 (38)
Non-Latino White	22 (10)
Other groups ^3^	32 (14)
**Monthly household income**	
≤$1000	34 (15)
$1001–$1500	29 (13)
$1501–$2000	42 (19)
>$2000	64 (28)
Don’t know/refused	56 (25)
**Maternal Education**	
Less than High School	47 (21)
High School/GED	72 (32)
Some college education or more	106 (47)
**Maternal Employment Status**	
Employed (full/part-time)	105 (45)
Unemployed	120 (55)
**Participating in WIC**	
Enrolled in WIC	189 (84)
Not Enrolled in WIC	36 (16)
**Participating in SNAP**	
Enrolled in SNAP	103 (46)
Not Enrolled in SNAP	122 (54)
**Milk-feeding type ^4^**	
Breastmilk only	36 (16)
Mixed—both breastmilk and formula	34 (15)
Formula only	154 (69)

^1^ Percentage decimals are rounded to the nearest whole number; ^2^ mothers reporting their race/ethnicity; ^3^ other groups include Asian, Pacific Islander, Mixed, and Native American; GED: general educational development; WIC: Special Supplemental Nutrition Program for Women, Infants, and Children; SNAP: Supplemental Nutrition Assistance Program; ^4^ milk-feeding type at the time of the interview or 9 month of infant’s age.

**Table 2:** Explanation of contribution of iron-fortified cereal and heme-iron sources to daily iron intake among infants at 9 months of age (*n* = 224).

Items	*n* (%)	Iron (mg) ^a^	General Description ^b^
**Infant cereal ^c^**	104 (46)	6.75 (3.38–13.50)	Rice, oats, and whole wheat with fruit flavors
**Animal source ^d^**	93 (41)	0.43 (0.18–0.67)	**--**
*Animal source categories*			
Infant meats ^e^	23 (10)	0.49 (0.30–0.67)	Chicken & gravy, turkey & gravy, apple & chicken.
Eggs	36 (16)	0.45 (0.29–0.60)	Boiled or omelet
Red meat	11 (5)	0.16 (0.07–0.85)	Ham, beef, hot dog
Poultry	45 (20)	0.24 (0.11–0.58)	Chicken or turkey (in non-infant meat variety)

^a^ Among infants who consumed that item: Median (IQR) contributions of iron from food sources, total daily iron intake. ^b^ Top three to four most consumed items within each category, accounting for 70% or more of the total variety; ^c^ iron-fortified; ^d^ categories of animal sources of iron consumed (meats and/or eggs on the day of recall); ^e^ includes all the varieties—chicken, turkey, ham and beef. Most items were stage 2 foods containing either a single meat ingredient or a combination of meat with fruits, vegetables or grains.

**Table 3:** Explanation of percentage of infants consuming iron-fortified formula, infant cereal, and meat/eggs, and their contribution to daily iron intake by milk-feeding type (*n* = 224).

**Milk-Feeding Type**			
	Breastmilk only 36 (16%)	Mixed 34 (15%)	Formula only 154 (69%)
**Mean (SD)**			
Average iron intake (mg)	7.21 (±8.03)	9.72 (±6.27) *	17.58 (±9.83) **
**Consumed ^a^**			
Infant cereal	33%	47%	49%
Meat and eggs	44%	47%	39%
**Contribution ^b^**			
Infant formula	-	47%	63%
Infant cereal	76%	52%	39%
Meat and eggs ^c^	9%	8%	4%

^a^ Number of infants (%) consumed that item at least once on the previous day; ^b^ Among those who consumed: the percentage of contribution towards total dietary iron intake; ^c^ refers to all types of infant jar meats and regular meat. ** Iron intake of the formula-only group was significantly greater compared to other feeding types (*p* < 0.001). * Iron intake of the mixed group was marginally greater compared to the breastmilk-only group (*p* = 0.77).

## Data Availability

The dataset used in the current study is available from the corresponding author upon reasonable request.

## Abbreviations

The following abbreviations are used in this manuscript:

BF	Breastfeeding Only
DGA	Dietary Guidelines for American
FF	Formula Feeding Only
FITS	Feeding Infants and Toddlers Study
MF	Mixed Feeding
NDSR	Nutrition Data System for Research
NHANES	National Health and Nutrition Examination Survey
U.S.	United States
WIC	Special Supplemental Nutrition Program for Women, Infants and Children

## References

[1] McCarthy E.K., Murray D.M., Kiely M.E. (2022). Iron Deficiency during the First 1000 Days of Life: Are We Doing Enough to Protect the Developing Brain?. Proc. Nutr. Soc..

[2] Medise B.E. (2021). The Role of Iron for Supporting Children’s Growth and Development. World Nutr. J..

[3] Gallahan S., Brower S., Wapshott-Stehli H., Santos J., Ho T.T.B. (2024). A Systematic Review of Isotopically Measured Iron Absorption in Infants and Children Under 2 Years. Nutrients.

[4] Georgieff M.K. (2023). The Importance of Iron Deficiency in Pregnancy on Fetal, Neonatal, and Infant Neurodevelopmental Outcomes. Int. J. Gynecol. Obstet..

[5] United States Department of Agriculture, United States Department of Health and Human Services (2025). Dietary Guidelines for Americans 2025–2030.

[6] Van Elswyk M.E., Murray R.D., McNeill S.H. (2021). Iron-Rich Complementary Foods: Imperative for All Infants. Curr. Dev. Nutr..

[7] Eldridge A.L., Catellier D.J., Hampton J.C., Dwyer J.T., Bailey R.L. (2019). Trends in Mean Nutrient Intakes of US Infants, Toddlers, and Young Children from 3 Feeding Infants and Toddlers Studies (FITS). J. Nutr..

[8] Davis K.E., Li X., Adams-Huet B., Sandon L. (2018). Infant Feeding Practices and Dietary Consumption of US Infants and Toddlers: National Health and Nutrition Examination Survey (NHANES) 2003–2012. Public Health Nutr..

[9] Hodges L., Toossi S., Todd J.E., Ryan-Claytor C. (2024). Special Supplemental Nutrition Program for Women, Infants, and Children (WIC): Background, Trends, and Economic Issues: 2024 Edition.

[10] Black M.M., Trude A.C.B., Armstrong B. (2019). Prenatal Special Supplemental Nutrition Program for Women, Infants, and Children Participation: A Step Toward Human Capital Development. JAMA Pediatr..

[11] U.S. Department of Agriculture WIC Food Packages. https://fns-prod.azureedge.us/sites/default/files/WICFoodPackageOptions.pdf.

[12] Guthrie J.F., Catellier D.J., Jacquier E.F., Eldridge A.L., Johnson W.L., Lutes A.C., Anater A.S., Quann E.E. (2018). WIC and Non-WIC Infants and Children Differ in Usage of Some WIC-Provided Foods. J. Nutr..

[13] Abrams S.A., Hampton J.C., Finn K.L. (2021). A Substantial Proportion of 6- to 12-Month-Old Infants Have Calculated Daily Absorbed Iron below Recommendations, Especially Those Who Are Breastfed. J. Pediatr..

[14] Hambidge K.M., Sheng X., Mazariegos M., Jiang T., Garces A., Li D., Westcott J., Tshefu A., Sami N., Pasha O. (2011). Evaluation of Meat as a First Complementary Food for Breastfed Infants: Impact on Iron Intake. Nutr. Rev..

[15] Jun S., Catellier D.J., Eldridge A.L., Dwyer J.T., Eicher-Miller H.A., Bailey R.L. (2018). Usual Nutrient Intakes from the Diets of US Children by WIC Participation and Income: Findings from the Feeding Infants and Toddlers Study (FITS) 2016. J. Nutr..

[16] Westrich B., Buzzard M., Gatewood L., McGovern P. (1994). Accuracy and Efficiency of Estimating Nutrient Values in Commercial Food Products Using Mathematical Optimization. J. Food Compos. Anal..

[17] R Core Team (2025). R: A Language and Environment for Statistical Computing.

[18] Donoghoe M.W., Marschner I.C. (2018). Logbin: An R Package for Relative Risk Regression Using the Log-Binomial Model. J. Stat. Softw..

[19] Donoghoe M. (2025). logbin: Relative Risk Regression Using the Log-Binomial Model.

[20] Bates M., Gupta P., Cogswell M., Hamner H., Perrine C. (2020). Iron Content of Commercially Available Infant and Toddler Foods in the United States, 2015. Nutrients.

[21] Klerks M., Bernal M.J., Roman S., Bodenstab S., Gil A., Sanchez-Siles L.M. (2019). Infant Cereals: Current Status, Challenges, and Future Opportunities for Whole Grains. Nutrients.

[22] Roess A.A., Jacquier E.F., Catellier D.J., Carvalho R., Lutes A.C., Anater A.S., Dietz W.H. (2018). Food Consumption Patterns of Infants and Toddlers: Findings from the Feeding Infants and Toddlers Study (FITS) 2016. J. Nutr..

[23] Nicklas T.A., O’Neil C.E., Fulgoni V.L. (2020). Nutrient Intake, Introduction of Baby Cereals and Other Complementary Foods in the Diets of Infants and Toddlers from Birth to 23 Months of Age. AIMS Public Health.

[24] Finn K., Quick S., Anater A., Hampton J., Kineman B., Klish W. (2022). Breastfed and Mixed Fed Infants Who Do Not Consume Infant Cereal Are at Risk for Inadequate Iron Intake:Data from the Feeding Infants and Toddlers Study 2016, a Cross-Sectional Survey. BMC Pediatr..

[25] American Academy of Pediatrics (2022). Where We Stand: Vitamin D & Iron Supplements for Babies.

[26] Dharod J.M., Hernandez M., Labban J.D., Black M.M., Ammerman A., Frazier C., Raynor N., Ramos-Castillo I. (2023). Associations between Early Introduction to Complementary Foods, Subsequent Cereal-Added Bottle Feeding and Daily Macronutrient Intake among Infants. Appetite.

[27] Clark K.M., Li M., Zhu B., Liang F., Shao J., Zhang Y., Ji C., Zhao Z., Kaciroti N., Lozoff B. (2017). Breastfeeding, Mixed, or Formula Feeding at 9 Months of Age and the Prevalence of Iron Deficiency and Iron Deficiency Anemia in Two Cohorts of Infants in China. J. Pediatr..

[28] Bailey R.L., Stang J.S., Davis T.A., Naimi T.S., Schneeman B.O., Dewey K.G., Donovan S.M., Novotny R., Kleinman R.E., Taveras E.M. (2022). Dietary and Complementary Feeding Practices of US Infants, 6 to 12 Months: A Narrative Review of the Federal Nutrition Monitoring Data. J. Acad. Nutr. Diet..

[29] Strzalkowski A., Black G., Young B.E. (2023). Iron and DHA in Infant Formula Purchased in the US Fails to Meet European Nutrition Requirements. Nutrients.

[30] Abrams S.A., Bergner E.M. (2023). Perspective: Is It Time to Revise the Current Nutrient Requirements for Infant Formulas Principally Established in 1980?. Adv. Nutr..

[31] Papanikolaou Y., Fulgoni V.L. (2018). Egg Consumption in Infants Is Associated with Longer Recumbent Length and Greater Intake of Several Nutrients Essential in Growth and Development. Nutrients.

[32] Werner E.R., Arnold C.D., Caswell B.L., Iannotti L.L., Lutter C.K., Maleta K.M., Stewart C.P. (2022). The Effects of 1 Egg per Day on Iron and Anemia Status among Young Malawian Children: A Secondary Analysis of a Randomized Controlled Trial. Curr. Dev. Nutr..

